# Case of Spina Bifida

**Published:** 1858-12

**Authors:** John M’Oscar


*Case of Spina Bifida.* By JOHN M'OSCAR, M. D.

I delivered Mrs. J., July 20, 1855, of a plump male child. There was a tumor the size of an orange over the first three lumbar vertebræ, composed chiefly of the spinal membranes, which blended gradually with the sound skin. The tumor was shiny and transparent. A slight concave Indian-rubber air pad, with an inflating nozzle to regulate its dimensions, was applied, and bound down by a strap bandage over the body. The tumor increased rapidly in size, which no regulated amount of pressure could subdue. At the end of a fortnight it was punctured, and about ℥iv. of spinal fluid drawn off. The pad was again applied; but on the following day it regained its previous size. At the end of another week it was again punctured, and about ℥vj. of fluid removed. No permanent good resulted; the tumor went on to increase for three weeks, when the child died, having lived six weeks in nearly constant pain and whining, which large doses of opium failed to relieve. The limbs throughout retained their motor power, but sensation was diminished. No convulsions supervened until just before death. No post-mortem. 4, Tyler-street, Regent-street.—*London Medical Times & Gazette.*

